# Association of impaired sensitivity to thyroid hormone with low bone mineral density and bone mineral content

**DOI:** 10.3389/fendo.2026.1845121

**Published:** 2026-07-30

**Authors:** Guoning Meng, Yuxian Bing, Yulin Liu, Zufei Zhou, Yuanmeng Li, Yichen Han, Yaqiu Jiang

**Affiliations:** 1 Department of Geriatrics, The First Affiliated Hospital of China Medical University, Shenyang, China; 2 Department of Endocrinology and Metabolism, Institute of Endocrinology, National Health Commission of the People's Republic of China Key Laboratory of Diagnosis and Treatment of Thyroid Diseases, The First Affiliated Hospital of China Medical University, Shenyang, China

**Keywords:** bone mineral content, bone mineral density, thyroid hormone, sensitivity to thyroid hormones, thyroid stimulating hormone

## Abstract

Osteoporosis is a systemic condition characterized by reduced bone mineral density (BMD) and bone mineral content (BMC), with its global prevalence increasing annually. Thyroid hormone (TH), as well as the sensitivity of central and peripheral target tissues to TH, play a regulatory role in bone health. This review examines traditional thyroid function indicators and thyroid hormone sensitivity indices, systematically evaluating their effects on BMD and BMC of different skeletal sites. Changed sensitivity of central and peripheral tissues to THs lead to distinct regulatory effects on bone mass. Sensitivity indices exhibit less variability and offer a more comprehensive reflection of thyroid hormone homeostasis, providing a more nuanced understanding of the complex relationship between fluctuations in thyroid hormone levels and decreased BMD at osteoporosis-prone sites, such as the vertebrae and femoral neck. This paper reviewed the relationship of classical thyroid function parameters, as well as central and peripheral thyroid hormone sensitivity indices, with decreased bone mass at osteoporosis-prone sites. While numerous published studies have supported an association between altered central and peripheral thyroid hormone sensitivity and reduced bone mass, inconsistencies remain in the literature. Further large-scale, high-quality randomized controlled trials (RCTs) are warranted to clarify the causal relationship between these factors.

## 1 Introduction

Osteoporosis is a systemic disease characterized by reduced bone mass, destroyed skeletal microstructure and increased skeletal fragility, which is prone to fracture (1). With the aging population, the global prevalence of osteoporosis is on the rise (2). There are reports indicating that the global prevalence of osteoporosis was 10.3% before 2002, and increased to 22.2% during the period from 2002 to 2021 (3). Bone mineral density (BMD), calculated as bone mineral content (BMC) divided by bone area, is widely used as a surrogate marker of bone mass and fracture risk (4).

Thyroid hormones exert direct effects on chondrocytes and osteoblasts, primarily through thyroid hormone receptor α in bone and cartilage, regulating bone formation and growth. Thyroid function is mainly reflected by the levels of free triiodothyronine (FT3), free thyroxine (FT4), and thyroid-stimulating hormone (TSH) in the serum. The interactions among TSH, FT4, and FT3 are closely linked and tightly regulated within the hypothalamic-pituitary-thyroid (HPT) axis, forming a complex feedback loop that ensures thyroid homeostasis. However, conventional thyroid function parameters may not fully capture the dynamic interactions within the HPT axis. Thyroid hormone sensitivity indices have therefore been proposed as more comprehensive markers of thyroid function to assess the complex interactions between FT3, FT4, and TSH. The thyroid hormone sensitivity index is divided into central and peripheral sensitivity indices. Central sensitivity is measured using the thyrotrophic thyroxine resistance index (TT4RI), thyroid-stimulating hormone index (TSHI), and the thyroid feedback quantile-based index (TFQI). Peripheral sensitivity is assessed using the free triiodothyronine to free thyroxine (FT3/FT4) (5).

This review aims to systematically synthesize current evidence on the associations between thyroid hormone sensitivity and reduced bone mineral density at sites including the spine and femur, while also considering conventional indicators of thyroid function. This review reveals the differential impacts of central and peripheral sensitivity indices on BMD and BMC, providing novel insights for the early identification and intervention of low bone mass and osteoporosis.

## 2 Literature search strategy

A comprehensive literature search was performed in the PubMed database for studies published from January 2005 to November 2025, using the following keywords: “thyroid hormone”, “thyroid hormone sensitivity index”, “BMD”, and “BMC”. All identified records were manually screened, and those not relevant to the topic were excluded.

## 3 Sensitivity to thyroid hormone

### 3.1 Periphrial thyroid hormone sensitivity

The focus of contemporary medical research has increasingly shifted from the assessment of absolute circulating hormone concentrations to the evaluation of tissue-specific hormone responsiveness. Accumulating evidence suggests that hormone resistance constitutes a shared pathophysiological mechanism underlying a range of chronic metabolic and endocrine disorders. Insulin resistance, for example, is a well-established central feature of type 2 diabetes mellitus and metabolic syndrome (6).Importantly, hormone resistance is not uniform across tissues. Variability in deiodinase activity, hormone transporter expression, receptor subtype distribution, and receptor density leads to differential thyroid hormone action among tissues (7–9). To better assess peripheral thyroid hormone sensitivity, the FT3/FT4 ratio is widely used as a biomarker in both clinical and research contexts (5). In peripheral tissues, the FT3/FT4 ratio reflects the efficiency of conversion from FT4 to the biologically active FT3, thereby serving as an indicator of thyroid hormone bioavailability (10, 11). The ratio is calculated by dividing FT3 (pmol/L) by FT4 (pmol/L). A higher FT3/FT4 ratio is generally indicative of enhanced peripheral thyroid hormone activity and increased tissue sensitivity to thyroid hormones (10).

### 3.2 Central thyroid hormone sensitivity

Central thyroid hormone sensitivity can be assessed using several indices, including the TT4RI,TSHI,TFQI and PTFQI, serving as quantitative markers of pituitary thyrotropic function and providing a more comprehensive interpretation of thyroid functional status than isolated hormone measurements (12, 13). TFQI, first proposed by Martin Laclaustra, quantifies the deviation between the expected and actual inhibitory effect of FT4 on TSH secretion (5). A negative TFQI value indicates higher pituitary sensitivity, meaning that the actual TSH is lower than the TSH expected under the inhibitory effect of fT4. Conversely, a positive TFQI value indicates lower pituitary sensitivity, meaning that the actual TSH is higher than the TSH expected under the inhibitory effect of fT4, or suggests the presence of central resistance to thyroid hormones (5). TSHI was calculated as ln TSH (mIU/L) + 0.1345 × FT4 (pmol/L) (12). TT4RI was calculated as FT4 (pmol/L) × TSH (mIU/L) (14). Higher TSHI or TT4RI represented lower central sensitivity to thyroid hormones. TFQI is derived from the empirical joint distribution of FT4 and TSH and has the advantage of avoiding extreme values in individuals with thyroid dysfunction (5). TSHI, often described as “TSH adjusted for FT4,” corrects for physiological TSH suppression and thereby allows a more accurate assessment of pituitary thyrotroph function (12).

### 3.3 Clinical relevance of thyroid hormone sensitivity indices

Despite growing interest in thyroid hormone sensitivity indices, their clinical applicability remains limited. Currently, there are no universally accepted cut-off values for indices such as TFQI, TSHI, and TT4RI, and they are typically analyzed as continuous variables in epidemiological studies. Regarding FT3/FT4, limited evidence from small-scale or population-specific studies has suggested a range of approximately 2.2–2.9 (with FT3 in pg/mL and FT4 in ng/dL); however, this has not been validated in large populations or incorporated into clinical guidelines (15).

In terms of clinical utility, although these indices have been associated with BMD in several studies, their ability to predict osteoporosis or fracture risk beyond conventional clinical parameters remains unclear. To date, most available evidence is derived from cross-sectional analyses, and prospective data evaluating their incremental predictive value are lacking.

## 4 The association between sensitivity to thyroid hormone indices and reduced BMD and BMC

### 4.1 peripheral thyroid hormone sensitivity

This section summarizes the associations between central and peripheral thyroid hormone sensitivity and BMD, BMC, and osteoporosis risk across different populations (**Table 1**). On the one hand, studies in euthyroid U.S. adults have shown that the FT3/FT4 ratio is inversely associated with spinal BMC, and exhibits a stable negative correlation with lumbar spine BMD (P < 0.05). These findings indicate that greater peripheral thyroid hormone sensitivity (reflected by a higher FT3/FT4 ratio) is associated with lower lumbar BMD and reduced bone density in this population (16, 17). On the other hand, in euthyroid patients with newly diagnosed type 2 diabetes mellitus (T2DM), a lower FT3/FT4 ratio has been associated with an increased risk of low bone mass and osteoporosis. This association appears to be more pronounced among individuals with good glycemic control (HbA1c < 7%), suggesting that, in middle-aged and elderly patients with well-controlled T2DM, a higher FT3/FT4 ratio may exert a protective effect against bone loss and osteoporosis (15). FT3/FT4 ratio has shown both protective and adverse associations with BMD across studies. This discrepancy may be attributed to differences in study populations (e.g., NHANES cohorts vs. Chinese patients with T2DM), sample size and metabolic status.

**Table 1:** Summary of studies investigating the association between thyroid hormone sensitivity indices and bone mineral density and bone mineral content.

Study (Population)	Study design	Main statistical results	Adjusted for confounders	Sex-related findings	Limitations
Wang et al., 2024 (16) (NHANES) Both sexes (adults ≥20y) (N = 1844)	Cross-sectional	FT3/FT4 was negatively associated with spinal BMC (near-linear relationship); no significant association between central sensitivity indices and BMD	Age, sex, BMI, calcium, smoking, alcohol, hormone use, physical activity	Both sexes included; sex adjusted, no stratified analysis	No causal inference; self-reported OVF (recall bias); incomplete adjustment for confounders
Li et al., 2022 (US males) Male-only(N = 3107)	Cross-sectional	FT3/FT4 and TFQI were negatively associated with lumbar spine BMD; TFQI also negatively correlated with total femur and femoral neck BMD, especially in normal BMI group	Age, BMI, smoking, alcohol, hypertension, diabetes, HbA1c, Ca, P	Male-only population; significant associations observed in males	Lack of data on anti-osteoporotic treatment; male-only population limiting generalizability; inability to infer causality
Wu et al., 2023 (19) (T2DM) Both sexes (men ≥50y & postmenopausal women) (N = 433)	Cross-sectional	U-shaped association between TFQI and osteoporosis risk (inflection point −0.29), suggesting both low and high sensitivity may increase risk	Sex, age, BMI, T2DM duration, HbA1c, HOMA-IR, 25(OH)D, FT3, FT4	Sex-stratified analysis; association stronger in females,>60y	Single-center design limiting generalizability; inability to infer causality; small sample size limiting statistical power; residual confounding (medication factors)
Liu et al., 2023 (18) (NHANES older adults) Both sexes (men ≥50y & postmenopausal women) (N = 3403	Cross-sectional	FT3/FT4 and SPINA-GD positively associated with BMD; FT4, TSHI, TT4RI, TFQI negatively associated with BMD, indicating impaired sensitivity linked to bone loss	Age, sex, BMI, Ca, smoking, P	Both sexes included; sex adjusted, no stratified analysis	Inability to infer causality; residual confounding (vitamin D, calcium supplementation, glucocorticoid use); lack of data on thyroid antibodies
Fang et al., 2025 (15) (T2DM) Both sexes (≥45y) (N = 350)	Cross-sectional	Higher FT3/FT4 was associated with lower risk of low bone mass; association significant only in HbA1c <7% group, suggesting glycemic status modifies effect	Age, sex, BMI, HbA1c, metabolic indicators	Both sexes included; sex adjusted, no stratified analysis	Inability to infer causality; single-center T2DM population limiting generalizability; small sample size limiting statistical power; residual confounding (vitamin D, absorption factors); incomplete assessment of bone metabolism markers

### 4.2 Central thyroid hormone sensitivity

Existing research suggests that impaired central sensitivity to thyroid hormones may be significantly associated with decreased BMD across multiple skeletal sites. A study by Li Zhiwei and colleagues found that in euthyroid adults from the United States, TFQI was significantly negatively correlated with BMD at key sites, including the lumbar spine, total femur, and femoral neck. Specifically, higher TFQI values were associated with lower BMD levels. This association remained robust after adjusting for various confounding factors (17). Further supporting this conclusion, the study by Zhong Xin showed that TSHI, TT4RI were also negatively correlated with BMD (18). Notably, in euthyroid patients with T2DM, TFQI exhibited a U-shaped non-linear relationship with the risk of osteoporosis, with particularly strong associations observed in subgroups such as females, those aged over 60, and individuals with a BMI of 25–30 kg/m² (19). However, the association between central thyroid hormone sensitivity indices and BMD or BMC remains controversial. For instance, a study in euthyroid adults in the United States found no significant correlation between sensitivity indices (such as TSHI, TT4RI, and TFQI) and BMC, nor between these indices and spinal BMD (16). Additionally, research by Liu and colleagues suggested that impaired thyroid hormone sensitivity in euthyroid individuals was associated with osteoporosis and fractures (18).

### 4.3 Classical thyroid function indices

Classical thyroid function indices include FT3, FT4, and TSH. The following section summarizes the associations between variations in these indices and BMD/BMC across different populations (**Table 2**).

**Table 2:** Summary of studies investigating the association between thyroid function parameters and bone mineral density, osteoporosis, and fracture risk.

Study (Population)	Study design	Main statistical results	Adjusted for confounders	Sex-related findings	Limitations
Noh et al., 2014 (22) (Korean women) Female-only (≥65y) (N = 756)	Cross-sectional	Higher normal TSH was positively associated with lumbar spine BMD and inversely associated with osteoporosis risk, suggesting protective effect of higher TSH	Age, BMI, menopause duration, HbA1c, FT4	Female-only; significant association observed	Inability to infer causality; residual confounding (vitamin D, PTH); limited thyroid and bone measurements (potential misclassification); self-reported data (recall bias);
Aubert et al., 2017 (24) Both sexes(N=61595)	Meta-analysis	Low-normal TSH (0.45–0.99 mIU/L) and higher FT4 were associated with increased fracture risk, indicating thyroid function within reference range influences fracture risk	Age, sex, BMI, smoking, diabetes, BMD	Both sexes included; sex adjusted in pooled analysis	Limited population representativeness (predominantly White); thyroid function measured only at baseline; residual confounding (nutrition, deiodinase activity, thyroid antibodies);
Song et al., 2025 (39) Both sexes (T2DM) (N = 563)	Cross-sectional	FT3 positively associated with BMD at multiple skeletal sites, suggesting higher FT3 may protect bone mass	Age, smoking, alcohol	Both sexes included; no stratified analysis	Small sample size; lack of BMC and bone area; insufficiently explored effects of hypoglycemic agents on bone metabolism.
van Rijn et al.,2014 (23) (peri-menopausal women) Female-only (peri-menopausal) (N = 1477)	Cross-sectional	Higher FT4 within reference range was negatively associated with lumbar spine BMD and increased risk of low BMD	Age, BMI, smoking, alcohol	Female-only; significant association observed	limited BMD assessment sites (lumbar spine only); incomplete thyroid and bone biomarkers; single measurement of thyroid hormones; potential confounding by non-thyroidal illness.

#### 4.3.1 TSH

Research has shown that even individuals with normal TSH levels may experience disturbances in bone metabolism (20). Kim et al. found that in postmenopausal women in Korea, low-normal TSH levels (within the reference range) were associated with reduced BMD in the lumbar spine and femoral neck (21). In euthyroid Korean women aged over 65, lower TSH levels within the normal range were associated with lower lumbar spine bone density and a higher potential risk of osteoporosis (22). Similarly, a study by van Rijn et al. revealed that among postmenopausal euthyroid women with T2DM, low-normal TSH levels were correlated with decreased BMD at the femoral neck and hip joint (23). Furthermore, other reports suggest that in euthyroid adults, TSH levels at the lower end of the reference range (0.45-0.99 mIU/L) are associated with an increased risk of hip fractures. These findings indicate that low-normal TSH may serve as a potential early warning sign for fracture risk in individuals within the normal thyroid function range (24). The association between low TSH levels and reduced BMD remains controversial. Recent Norwegian studies found no significant correlation between normal serum TSH levels and BMD at the hip or forearm (25, 26). Similarly, a Taiwanese study reported no significant relationship between normal serum TSH and wrist BMD in euthyroid middle-aged adults (27). The inconsistencies in findings regarding the lower limit of the TSH reference range and low BMD may stem from three main sources: (1) heterogeneity in study populations (e.g., age, sex, comorbidities); (2) geographic variations, reflecting differences in environmental and dietary factors; and (3) variability in BMD measurement sites, as skeletal regions differ in sensitivity to TSH changes.

#### 4.3.2 FT3, FT4

A large-scale meta-analysis of prospective cohort studies has shown that, among individuals with normal thyroid function, elevated FT4 levels—despite remaining within the normal reference range—are significantly associated with an increased risk of hip fractures, any fractures, and non-vertebral fractures. This study suggests that higher-normal FT4 levels may serve as an independent early warning factor for fracture risk (24). This finding is further supported in the peri-menopausal female population, where research has confirmed that, after adjusting for confounding factors such as age, BMI, and smoking, low BMD in this group is independently associated with high-normal FT4 levels. Therefore, clinical assessments of bone health in peri-menopausal women should particularly focus on FT4 levels, with increased vigilance for the potential bone loss risks associated with FT4 levels at the upper end of the normal range (23).

Overall, current evidence on thyroid function parameters and bone outcomes remains inconsistent, likely reflecting differences in study design, populations, and bone assessment methods. Further prospective studies are needed.

## 5 Discussion

This study focuses on classic thyroid function parameters and thyroid sensitivity indices, analyzing their impact on BMD. Overall, the included studies suggest that thyroid hormone sensitivity indices and thyroid function parameters are significantly associated with BMD, BMC, and osteoporosis risk, even within the euthyroid range, Although the direction and magnitude of these relationships vary across studies.

Several cross-sectional studies have shown significant associations between TSHI, TT4RI, and the incidence of chronic kidney disease, non-alcoholic fatty liver disease (NAFLD), and coronary artery disease (CAD) (28–30). Other studies have reported that TFQI is closely associated with mortality from obesity, metabolic syndrome, and diabetes (5). Diabetes, obesity, metabolic syndrome, and NAFLD are all known risk factors for osteoporosis. Notably, impaired thyroid hormone sensitivity is closely linked to these conditions, which may partially explain the association between reduced thyroid sensitivity and decreased bone mass.

The dual association between the FT3/FT4 ratio and BMD may be explained by differences in study populations and metabolic status. The former studies included 3,107 euthyroid U.S. men, whereas the latter involved 350 newly diagnosed euthyroid Chinese patients with T2DM (≥45 years), with data derived from a single-center inpatient cohort, potentially introducing selection bias and limiting generalizability. In addition, subgroup analyses showed that the protective association was evident only in individuals with HbA1c < 7%. In T2DM, thyroid hormone profiles are often characterized by reduced FT3 levels due to impaired peripheral conversion of T4 to FT3, possibly related to chronic hyperglycemia, insulin resistance, inflammation, and oxidative stress (31). These alterations may represent an adaptive response to metabolic stress and could modify the relationship between thyroid function and bone health.

**Figure 1:**
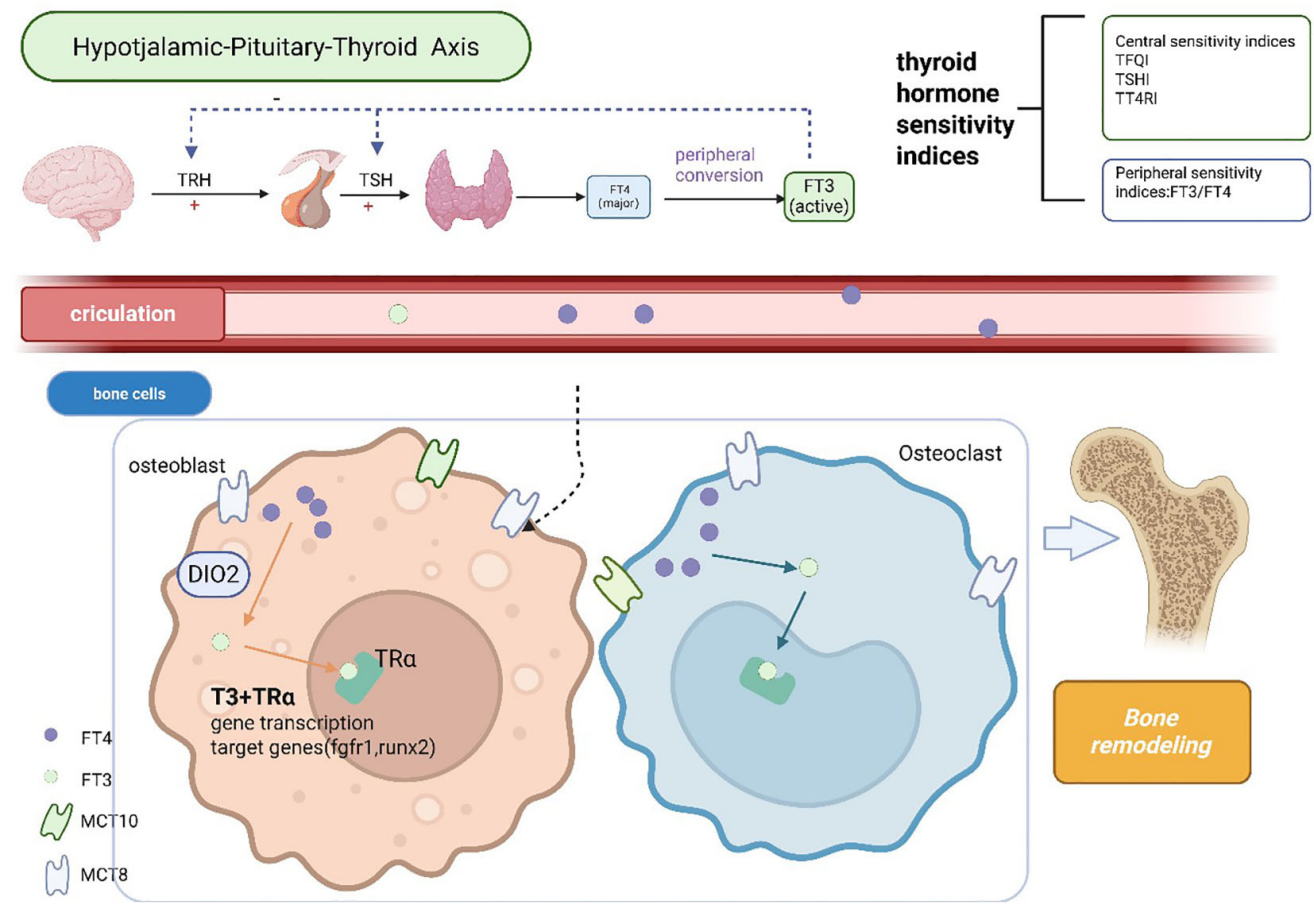
Circulating FT4 enters osteoblasts and osteoclasts via MCT8/MCT10 and is locally converted to T3 by DIO2. T3 binds to TRα1 to regulate gene transcription and bone remodeling. Central (TSHI, TT4RI, TFQI) and peripheral (FT3/FT4 ratio) thyroid hormone sensitivity indices are indicated.

The association between low BMD and higher levels of FT4 within the normal reference range can be explained by the direct regulatory role of thyroid hormone on bone metabolism. FT4 must be converted into active T3 in bone tissue by type 2 iodothyronine deiodinase (DIO2), a process essential for optimal bone strength and mineralization (32). T3 acts primarily by binding to THRα1, which is highly expressed in bone and mediates the dominant bone metabolic effects of thyroid hormone. (**Figure 1**). This accelerates osteoclast-mediated bone resorption and shortens the bone remodeling cycle, disrupting the coupling between bone formation and resorption and ultimately leading to bone loss (33). These mechanisms suggest that, in contrast to the tightly feedback-regulated TSH or the already-activated FT3, even subtle fluctuations in FT4 may be amplified through local DIO2 activity in bone, exerting long-term cumulative effects on bone metabolism. This explains why FT4 levels at the upper end of the normal range increase the risk of bone loss (23).

Several biological mechanisms may explain the interaction between thyroid function and bone metabolism. Thyroid hormones directly regulate bone remodeling by acting on osteoblasts, osteoclasts, and chondrocytes, thereby influencing both bone formation and resorption (33). Experimental evidence indicates that type 2 iodothyronine deiodinase (DIO2), expressed in osteoblasts, converts T4 into bioactive T3, thereby controlling local thyroid hormone availability independent of serum concentrations. The biological actions of T3 in bone are primarily mediated through thyroid hormone receptor alpha-1 (TRα1), the predominant receptor isoform expressed in osteoblasts and chondrocytes (34). Although both TRα and TRβ are present in skeletal cells, the expression level of TRα1 is approximately tenfold higher than that of TRβ1 (35). T3 binds to TRα1 and regulates the transcription of downstream target genes involved in osteoblast differentiation and bone formation (36). Consistent with this, experimental studies have shown that DIO2 deficiency in osteoblasts leads to reduced expression of fibroblast growth factor receptor 1 (Fgfr1), accompanied by a 37–52% decrease in bone formation rate and impaired bone strength (32).

Sex-specific differences in these associations may be largely driven by estrogen status. Estrogen deficiency disrupts bone homeostasis not only by directly inhibiting bone formation and promoting bone resorption, but also by inducing osteoimmune imbalance. It leads to dysregulation of CD4^+^ T cells and increased production of pro-inflammatory cytokines, including TNF-α, IFN-γ, IL-17, and RANKL. RANKL promotes osteoclast differentiation and activation, while TNF-α impairs osteoblast function by inducing autophagy and apoptosis. Furthermore, enhanced Th17 cell activity and suppressed regulatory T cell (Treg) function further exacerbate bone loss (37). Beyond the influence of sex hormones, additional endocrine regulators—particularly the vitamin D–parathyroid hormone (PTH) axis—may further modulate bone metabolism and interact with thyroid hormone signaling. Upon binding of calcitriol, activation of the vitamin D receptor (VDR) promotes osteoblast differentiation, a tightly regulated process involving the transition of mesenchymal stem cells into mature osteoblasts. VDR activation upregulates key osteogenic markers, including runt-related transcription factor 2 (Runx2), osterix (Osx), and alkaline phosphatase (ALP), which are essential for osteoblast maturation and subsequent bone matrix synthesis and mineralization (38).

Despite extensive research supporting the notion that bone metabolism can be disrupted even within the normal range of thyroid function, alterations in both peripheral and central thyroid hormone sensitivity have been associated with reductions in BMD and BMC in regions such as the spine and femur. However, recent studies have presented different viewpoints. For instance, Zhao et al. found that in euthyroid patients with type 2 diabetes, TFQI and TT4RI were not significantly associated with BMD at the lumbar spine, femoral neck, or hip (39). This seemingly contradictory finding highlights the variability in how changes in thyroid hormone sensitivity, both centrally and peripherally, affect bone metabolism across different populations, metabolic states, and skeletal sites. The specific mechanisms underlying these discrepancies remain unclear, and future research should aim to elucidate the mechanisms behind these conflicting observations.

Most included studies were cross-sectional in design, which limits causal inference and precludes determination of temporal relationships between thyroid-related indices and bone outcomes. This methodological limitation weakens the ability to establish directionality in the observed associations.

Although most studies adjusted for key variables such as age, sex, and BMI, the selection of additional covariates varied considerably across studies. Some studies further accounted for metabolic factors (e.g., HbA1c and lipid profiles), lifestyle factors (e.g., smoking, alcohol consumption, and physical activity), and bone-related biomarkers. Notably, although sex was commonly included as a covariate, sex-stratified analyses were rarely performed, limiting the ability to fully elucidate sex-specific differences. In addition, vitamin D status and parathyroid hormone levels are also key determinants of bone metabolism and may act as important confounders in this context. The inconsistent adjustment for these variables across studies may contribute to residual confounding and between-study heterogeneity.

Current research has several significant limitations. First, most studies have not collected data on participants’ history of anti-osteoporotic treatments (such as bisphosphonates, calcium supplements, etc.) or included key bone metabolism markers, such as 25-hydroxyvitamin D and PTH, which could lead to confounding bias. Second, the majority of existing studies are cross-sectional and lack follow-up data. Given these limitations, future research should: 1) record participants’ history of anti-osteoporotic treatments and include key regulators of bone metabolism to control for potential confounding;2) conduct well-designed prospective cohort studies and RCT with repeated measurements to elucidate the causal relationship between thyroid hormone sensitivity and bone health;3) include euthyroid individuals, postmenopausal women, and patients with metabolic disorders such as T2DM as target populations, given their higher susceptibility to bone loss;4) incorporate comprehensive indices of thyroid hormone sensitivity, TSHI, TT4RI, TFQI, and FT3/FT4 ratio, to better capture central and peripheral thyroid regulation.

## 6 Conclusion

In summary, central and peripheral thyroid sensitivity indices, compared to traditional thyroid function parameters, exhibit smaller deviations and are more reliable in reflecting the regulation of TH homeostasis. While numerous studies support the association between changes in thyroid hormone sensitivity and decreased bone mass, inconsistencies remain in the literature. Large-scale, high-quality randomized controlled trials are warranted to clarify the causal relationship between thyroid hormone sensitivity and bone loss.
